# BTK inhibitors for Severe Acute Respiratory Syndrome Coronavirus 2 (SARS-CoV-2): A Systematic Review

**DOI:** 10.21203/rs.3.rs-319342/v1

**Published:** 2021-03-22

**Authors:** Michael Stack, Keith Sacco, Riccardo Castagnoli, Alicia A. Livinski, Luigi D. Notarangelo, Michail S. Lionakis

**Affiliations:** Mayo Clinic Florida; Mayo Clinic Florida; Mayo Clinic Florida; National Institutes of Health; Mayo Clinic Florida; NIAID, NIH, Bethesda

**Keywords:** Acalabrutinib, Bruton’s tyrosine kinase, Acute respiratory distress syndrome, COVID-19, Cytokine storm, Hospitalization, Ibrutinib, SARS-CoV-2, X-linked agammaglobulinemia

## Abstract

**Importance:**

The Bruton tyrosine kinase (BTK) regulates B cell and macrophage signaling, development, survival, and activation. BTK inhibition was shown to protect against lethal influenza-induced acute lung injury in mice. Inhibiting BTK has been hypothesized to ameliorate lung injury in patients with severe coronavirus disease 2019 (COVID-19).

**Objective:**

To evaluate the use of BTK inhibitors (BTKinibs) during COVID-19 and assess how they may affect patient outcomes.

**Evidence Review:**

We searched PubMed, Embase, and Web of Science: Core on December 30, 2020. Clinical studies with at least 5 COVID-19 patients treated with BTKinibs were included. Case reports and reviews were excluded.

**Findings:**

One hundred twenty-five articles were identified, 6 of which met inclusion criteria. Sample size ranged from 6 to 126 patients. Patient populations included subjects hospitalized with COVID-19 (6/6) and admitted to the intensive care unit (5/6). Patient age ranged between 35 and 98 years. Four studies included patients already receiving BTKinibs for their lymphoproliferative disease, 1 for Waldenstrom’s macroglobulinemia and 3 for chronic lymphocytic leukemia (CLL). The most common clinical outcomes measured were oxygen requirements (4/6) and hospitalization rate or duration (3/6). Differences in standard-of-care reflected the date of study and pre-existing conditions in the various patient cohorts. Full-dose acalabrutinib was evaluated in 2 studies, one study evaluated full-dose ibrutinib, and another study evaluated both ibrutinib and acalabrutinib. The remainder 2 studies described outcomes in CLL patients on multiple BTKinibs and other CLL-targeted treatments. Three studies showed decreased oxygen requirements in patients who started or continued BTKinibs. All three studies that evaluated hospitalization rate or duration found favorable outcomes in those on BTKinibs.

**Conclusions and Relevance:**

BTKinib use was associated with decreased oxygen requirements and decreased hospitalization rates and duration. However, randomized clinical trials are needed to validate the beneficial effects of BTKinibs for acute SARS-CoV-2 infection.

## Introduction

The severe acute respiratory syndrome coronavirus 2 (SARS-CoV-2) is the causative agent of coronavirus disease 2019 (COVID-19) that rapidly spread into a global pandemic causing >2.5 million deaths worldwide [1]. The standard-of-care for COVID-19 has evolved over the past year alongside the continued elucidation of pathogenetic mechanisms and the identification of biomarkers differentiating asymptomatic from severely-ill individuals. Such immune parameters include elevated inflammatory cytokines/chemokines (including IL-1β, IL-6, G-CSF, GM-CSF, MCP-1) and a skewed neutrophil to lymphocyte ratio [2–4]. We recently showed that COVID-19 patients had elevation of certain immune biomarkers associated with activation of the innate immune system and greater mortality [5]. The COVID-19-associated hyperinflammatory response has features that resemble macrophage activation syndrome, marking signals within the innate immune system as potentially targetable for treatment [6]. The Bruton’s tyrosine kinase (BTK) has recently been proposed as one of those targets. Indeed, blood monocytes from patients with severe COVID-19 showed increased BTK activation and production of interleukin-6 correlating with systemic inflammation [7].

BTK is essential for B cell development within the bone marrow. X-linked agammaglobulinemia stems from germline loss-of-function mutations in *BTK,* resulting in a block of B cell development starting at the pro-B cell stage, with absence of peripheral B cells [8]. BTK plays a critical role in the proliferation and survival of leukemic B cells [9]. Consequently, BTKinibs such as ibrutinib and acalabrutinib have been successfully used to treat patients with CLL and Waldenstrom’s macroglobulinemia (WM) [10, 11]. Not limited to its effects on B cells, BTK has been coined “an emerging key player in innate immunity” [12]. Studies have described roles for BTK in multiple TLR signaling pathways, TREM-1, and interferon (IFN) production [13–16]. Many of these pathways, including the BTK-dependent activation of NF-B, have been implicated in hyperinflammation during severe COVID-19 [17]. As mentioned earlier, severe COVID-19 patient monocytes have significantly elevated BTK phosphorylation compared to healthy volunteers [7]. As the role of BTK in cells of the myeloid lineage continues to be elucidated, use of BTKinibs has been expanded beyond B cell malignancies. For example, ibrutinib has demonstrated a protective role against lethal influenza- and lipoteichoic acid-induced lung injury in mice, including the reduction of the inflammatory cytokine IL-6 [18, 19]. Concurrent with the finding that neutrophilic expression of several granule proteins (myeloperoxidase, elastase, gelatinase) is BTK-dependent, CLL patients on ibrutinib had reduced neutrophil degranulation and rapid reduction of oxidative burst [20–22], which may account for the heightened risk of some BTKinib-treated patients to opportunistic fungal infections [23]. Other important roles recently observed include a possible role for BTK in NLRP3 inflammasome activation [24].

Improved therapeutics are necessary to combat the significant morbidity and mortality from SARS-CoV-2 infection, and off-label drugs have bolstered the repertoire of available treatments. Nonetheless, off-label medication use must be reviewed to describe a tangible result in the clinic while clinical trials are still ongoing. Notwithstanding the presently narrow clinical indications of BTKinibs, the connectedness of factors affected by severe COVID-19 and BTK signaling makes BTKinibs attractive therapeutic candidates for patients with severe SARS-CoV-2 infection. It has been hypothesized that BTKinibs can ameliorate the hyper-inflammatory response in COVID-19 and improve survival [25].While few studies have reported on the use of BTKinibs in patients with COVID-19, it is unclear whether their use is associated with robust improvement in pre-specified clinical outcomes. To this end, we undertook a systematic review aimed to describe clinical outcomes measured from BTKinib use during acute SARS-CoV-2 infection.

## Methods

We used the Preferred Reporting Items for Systematic Reviews and Meta-Analyses (PRISMA) Checklist for reporting our review [26].

### Eligibility Criteria

#### Types of Studies

We included case-cohort studies, interventional cohort studies, and randomized controlled trials. Clinical studies with at least 5 patients were included. Individual case reports and reviews were excluded. A confirmed PCR diagnosis of SARS-CoV-2 infection, whether leading to symptomatic or asymptomatic infection, was required for inclusion. Studies of both hospitalized and ambulatory patients were included. There were no age, gender or ethnicity exclusions. Only studies published in English in 2020 were included.

#### Types of Interventions

We included both studies in which BTKinibs were used as off-label treatment for acute SARS-CoV-2 infection and studies where patients were already receiving BTKinibs for other indications (primarily lymphoproliferative disorders) and in which BTKinibs were continued during the course of acute SARS-CoV-2 infection. There were no exclusions for BTKinib dosing limits.

#### Types of Outcome Measures

We included all clinical outcomes described following a diagnosis of acute SARS-CoV-2 infection. These included adverse outcomes related to BTKinib administration, rate and duration of hospitalization, escalation of care including admission to intensive care unit (ICU), increased oxygenation requirements including need for mechanical ventilation, and overall survival.

#### Information Sources and Searching

A medical research librarian (A.L.) developed and ran searches on December 30, 2020 in the following databases: PubMed (US National Library of Medicine), Embase (Elsevier), and Web of Science: Core Collection (Clarivate Analytics). Search terms used were keywords and controlled vocabulary terms (e.g., MeSH in PubMed and EMTREE in Embase) for each concept of interest (see Supplemental File 1 for final search strategies).

The reference lists of abstracts, conference proceedings, and included articles were also reviewed by authors to identify additional studies that had not been identified by database searches.

### Study Selection

Two reviewers (M.S. and K.S.) independently reviewed the titles and abstracts using the established eligibility criteria and piloted screening forms. EndNote (Clarivate Analytics) and Microsoft Excel were used to screen the search results. Disagreements were resolved by consulting with a third reviewer (R.C.). The full-text of the articles included at the end of title and abstract screening were independently screened by two reviewers (M.S. and K.S.) to assess whether they met the predetermined inclusion and exclusion criteria. Reasons for exclusion were documented at this stage.

### Data Collection and Analysis

We conducted a qualitative analysis of each study and their clinical outcomes as described. Microsoft Excel was used for the data collection and analyses. Two reviewers (M.S. and K.S.) assessed the risk of bias in the articles included using the the Cochrane Collaboration’s RoB 2.0: Risk-Of-Bias Tool for Randomized Trials [27], and the adapted Newcastle-Ottawa Scale (NOS) for cohort studies and case-control studies as per reference [28]. We evaluated as to whether we could perform a pooled cohort analysis of the same clinical outcome described in multiple studies; however, we deferred this analysis due to significant heterogeneity in population cohorts, drug dose, duration, and different approaches to the defined standard-of-care at the time of the studies.

## Results

**Figure 1:**
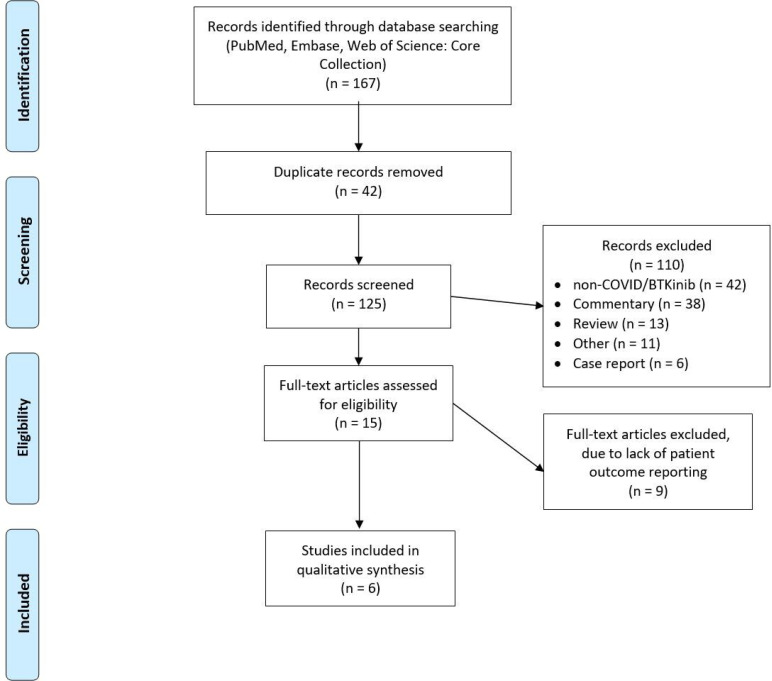
Flow Diagram of Inclusion of Studies in our Analysis.

Our literature search retrieved 167 articles from the three databases of which 42 were duplicate articles and 125 represented unique articles. One hundred ten articles were excluded at the title and abstract stage due to various reasons [review/commentary (n=51); non-COVID-19-related use of BTKinibs (n=42); other reasons (n=11); case reports (n=6)]. The 15 remaining articles were screened at the full text screening stage. Among those, 9 were excluded as no patient outcomes were reported and 6 articles were included in our analysis (Figure 1).

The sample size ranged from 6 patients, in a case-series of WM [29] to 126 subjects in an ongoing phase II randomized trial [30]. The majority of the population consisted of patients who were hospitalized with acute COVID-19, and included patients requiring various modalities of oxygenation support and ICU admission (Table 1). The median ages of the patients in each study ranged from 61 to 72 years. The cohorts included patients with WM and CLL, as they are commonly treated with BTKinibs. Two studies focused on the use of acalabrutinib [7, 30], one on ibrutinib [29], and another evaluated the effect of either drug [31]. Two other studies did not separately classify patients on the basis of which BTKinib was used (i.e., acalabrutinib, ibrutinib, or zanubrutinib), nor included the specific dosages of each drug [32, 33]. The 6 studies had a heterogeneity of clinical outcomes measured, including resolution of symptoms, hospitalization rate or duration, oxygen requirements, and overall survival (Table 1). Time to primary outcome measurement varied from 12 days to 30 days. Of note, the study by Wilkinson et al. was ongoing at the time of analysis, therefore no outcome data is available thus far [30].

Hospitalization was a key outcome measured in half of the studies. Scarfò et al. reported on 190 CLL patients with confirmed SARS-CoV-2 infection, and found that ibrutinib significantly lowered the hospitalization rate for severe COVID-19 compared to any other CLL-targeted treatment or no CLL-directed treatment by half (OR 0.44 [95% CI 0.20–0.96] p<0.05) [32]. Thibaud et al. measured hospitalization duration, as all eight patients on BTKinib were hospitalized. Patient groups were stratified to BTKinib that was continued (n=6) or discontinued (n=2) during acute COVID-19. The median length of stay was 6 days (range, 3–9) for the group that continued BTKinibs, and 8.5 days (range, 5–19) for the group in which use of BTKinibs was discontinued [31].

Three studies reported outcome data related to improvement in oxygenation requirements. Roschewski et al. reported on 19 patients hospitalized with COVID-19, in whom acalabrutininb was administered off-label [7]. Eighteen of these patients had increasing oxygen requirements at baseline, 11 requiring supplemental oxygen without intubation and 8 requiring mechanical ventilation. Eight out of 11 (73%) subjects requiring supplemental oxygen without intubation were discharged from the hospital on room air at the end of the 10–14-day treatment period. Four out of 8 (50%) subjects on mechanical ventilation were extubated in the same time period, including 2 (25%) who were discharged from the hospital on room air [7]. Mato et al. reported that hospitalized CLL patients with COVID-19 who were receiving BTKinib therapy appeared to require supplemental oxygen (86% versus 92%) or mechanical ventilation (21% versus 30%) less frequently compared to those not receiving BTKinibs [33]. In addition, the patient group that discontinued BTKinibs in the Thibaud et al. study had higher rates of oxygen requirement, with 4/6 (67%) requiring nasal cannula or more aggressive oxygenation support. Of the 2 patients who continued ibrutinib in that study, one required minimal oxygenation support via nasal cannula [31].

In a case series of 6 WM patients, Treon et al. observed that ibrutinib continuation favored resolution of COVID-19 symptoms [29]. The median time with COVID-19-related symptoms prior to diagnostic testing was 5 days, whereas the median time since diagnosis (i.e., time of data collection) was 22 days. Five of the 6 subjects continued full-dose ibrutinib (420mg/day) throughout their acute SARS-CoV-2 infection. Among these, only one subject had unresolved symptoms at day 24 since diagnostic testing. In contrast, ibrutinib was held in the sixth subject. Because symptoms worsened, ibrutinib was restarted at 140mg/day on hospital day 5, along with tocilizumab. The subject was intubated on day 10. Ibrutinib was increased to full-dose (420mg/day) on day 11, which was associated with a rapid improvement in oxygenation. The patient was extubated on day 12 and discharged from the hospital on room air by day 14.

Overall survival/mortality was measured in 5 of 6 studies (Table 1). The Treon et al. study reported that all 6 WM patients survived SARS-CoV-2 infection [29]. Scarfò et al. reported that 13/31 (41.9%) patients with severe COVID-19 died while receiving ibrutinib. One of these 13 (7.7%) patients with less severe COVID-19 died while receiving ibrutinib. Another 42 out of 146 (28.8%) patients not receiving BTKinibs died with COVID-19 in that study, however it is unclear as to which other CLL-targeted treatment was continued or discontinued in this group and what proportion of these patients had severe versus less severe COVID-19. In addition, the study did not report an overall survival between patients who remained on ibruritinib versus those in whom ibruritinib was discontinued [32]. Roschewski et al. reported 5/19 deaths from COVID-19, one out of 11 (9%) in the supplemental oxygen group not requiring intubation and four out of 8 (50%) in the mechanical ventilation group [7]. Mato et al. reported that patients in the BTKinib group trended towards higher survival when compared to those on other or no CLL-directed treatment, but the difference was not statistically significant (HR 0.80, 95% CI [0.47–1.4], p=0.42) [33]. Thibaud et al. reported that both subjects who continued BTKinib therapy survived, while 2 out of 6 patients (33%) in whom BTKinibs were discontinued died [31].

We did not perform a pooled cohort analysis from the studies identified due to the study population heterogeneity and multiple biases that were identified during the risk-of-bias analysis (Table 2). Indeed, selection and treatment biases were significant among cohorts studies. Further, variable definitions of clinical outcomes would not have enabled representative pooled outcomes.

## Discussion

The ongoing COVID-19 pandemic has shown that significant morbidity and mortality is associated with systemic hyper-inflammation, whereby innate immune cells including activated monocytes/macrophages appear to contribute to pathologic inflammation [34]. From a mechanistic standpoint, BTKinibs could suppress myeloid cell activation and prevent acute lung injury which is a major contributing factor for mortality in COVID-19 [2]. As is the case with a number of off-label therapeutics used to treat SARS-CoV-2 infection, questions arise as to drug dosing, duration, and timing during the course of the disease. Our review identified 6 studies with defined clinical outcomes pertaining to the use of BTKinibs during acute SARS-CoV-2 infection. A majority of the patients in these 6 studies had underlying lymphoproliferative disorders (CLL or WM) and were receiving BTKinibs to treat their underlying malignancy when they developed COVID-19. This may skew data towards decreased survival as COVID-19 appears to be more severe in patients with CLL, with a reported fatality rate of 37% among subjects requiring COVID-19-related hospitalization [35].

The main outcomes measured in the published studies related to hospitalization and ICU admission rates, hospitalization duration, and oxygenation requirements. While these are important outcome measures particularly during increased rates of community spread and infection, these studies did not currently identify use of BTKinibs to be associated with improved overall survival. The Open-label Randomized CALAVI (NCT04346199) study enrolled 177 participants to assess the effect of acalabrutinib as compared to standard-of-care in hospitalized patients with COVID-19 [36]. The study reportedly failed to meet the primary endpoint of overall survival and no respiratory failure at day 14 and was terminated, although no official published data is available for analysis as of February 21, 2021 [37]. It has been hypothesized that a combination of antivirals and anti-inflammatory agents may be required to significantly improve overall survival [38]. The antiviral drug remdesivir was associated with a more rapid recovery from COVID-19 but did not confer a statistically significant survival benefit [39]. Anti-inflammatory therapeutics have shown a survival benefit and/or hastening in patient recovery in hospitalized patients with COVID-19. The use of dexamethasone (6mg daily for up to 10 days) led to lower 28-day mortality in hospitalized patients receiving oxygenation support [40]. The anti-IL-6 receptor-targeted monoclonal antibody tocilizumab has shown promising clinical outcomes in hospitalized COVID-19 patients. Non-mechanically ventilated patients hospitalized with COVID-19 pneumonia who received two doses of tocilizumab 8 mg/kg had a lower likelihood of progression to a composite outcome of mechanical ventilation or death [41]. However, there was no improvement in overall survival. Conversely, the use of both IL-6 receptor antagonists tocilizumab and sarilumab led to improved 90-day surivival in ICU patients requiring organ support [42]. The Janus kinase (JAK) inhibitor baricitinib was recently showed to hasten recovery when used in combination with remdesivir compared to remdesivir monotherapy, but did not improve patient survival[43]. Further supportive clinical studies of anti-inflammatory therapeutics are needed. Of note, the emerging SARS-CoV-2 variants are less likely to decrease the therapeutic efficacy of anti-inflammatory drugs targeting dysregulated host defenses, which may be seen with certain monoclonal antibodies directed against the spike protein of SARS-CoV-2.

Our systematic review failed to identify robust outcome data from prospective, blinded, and randomized studies which represent the highest levels of evidence. Thus, our study had a number of limitations. Primarily, a significant proportion of acute COVID-19 patients were already receiving BTKinibs for treatment of their underlying lymphoproliferative disorders. Hence, there was a high risk of selection bias as patients were already on BTKinib treatment, or there was an absence of a comparative intervention in some of these studies. No blinding was performed in any study due to the absence of comparative groups. Only the ongoing Wilkinson et al. trial [30] had a low risk of detection bias because the primary outcome measured was resolution of symptoms in which the care provider is the outcome assessor. Complete data (<5% lost to follow-up) existed for the majority of included studies, with the exception of the ongoing ACCORD clinical study [30]. Non-random treatment discontinuation is an additional significant bias among studies in this systematic review. A majority of patients (35/44) in Scarfò et al. had ibrutinib treatment discontinued during acute COVID-19. Since ibrutinib is an irreversible BTKinib, it is possible that ibrutinib’s pharmacodynamic effects persisted in the discontinuation group and could have affected patient outcomes during acute SARS-CoV-2 infection [44].

Possible downsides to BTKinib use in acute COVID-19 should be considered. BTK is a key mediator of B cell activation, therefore its inhibition may affect antibody production during acute SARS-CoV-2 infection. However, Roeker et al. reported no significant association between ongoing use of BTKinibs and development of SARS-CoV-2 specific antibodies in 6 patients with CLL [45]. Sun et al. found that serum IgG levels did not significantly decrease in short term ibrutinib treatment (<12 months) for CLL. Surprisingly, IgA and IgM increased significantly after ibrutinib treatment [46]. This is likely due to deletion of clonal CLL cells and reconstitution of nonclonal B-cell precursors in the bone marrow with normalization of nonclonal B-cell numbers in peripheral blood [46, 47]. While the effect of BTKinibs on antibody production in healthy individuals has yet to be evaluated, it is interesting to observe that the few patients with X-linked agammaglobulinemia who developed COVID-19, manifested a relatively mild course of infection [48, 49], possibly implying a compensatory role of T cell-mediated immunity.

While BTK inhibition may supress the excessive inflammation caused by the innate immune response against SARS-CoV-2, it may also increase the risk for secondary infections. Fungal and other opportunistic infections such as pneumonia have been observed in CLL and other patients on ibrutinib [23, 50–52]. However concomitant use of CD20-depleting agents may confound such a risk since CD20-depleting agents are also associated with an increased risk of pneumonia [53]. Secondary infections have been observed in high incidence and implicated in worsening health outcomes among acute COVID-19 [54–56]. Therefore, any treatment increasing the risk of secondary infection should be administered with much caution.

Our systematic review highlights that trial data on BTKinib use in acute COVID-19 is currently lacking or pending completion. While there are trends for decreased oxygenation requirements and/or decreased hospitalization or care escalation, these results are based on a small number of studies of which most have a small sample size and describe patients receiving BTKinibs for alternative indications without comparative patient groups. However, the fact that patients already receiving BTKinibs may have improved clinical outcomes suggest that BTKinib use early in the course of SARS-CoV-2 infection may have a greater effect on clinical outcomes, as appears to be the case with Remdesivir or IFN-beta administration [57–59]. Further, as long-term complications of COVID-19 are being described, BTKinib use during the acute disease stage could affect the risk and/or severity of developing inflammatory complications such as cardiomyopathies and lung fibrosis. While the available data are not currently robust enough to recommend BTKinibs on a clinical basis for the treatment of acute SARS-CoV-2 infection, they do provide a framework to assess specific and longitudinal clinical outcomes associated with uncontrolled inflammation in COVID-19. The completion and reporting of randomized clinical trials of various BTKinibs in patients with COVID-19 will help elucidate the precise role of these targeted drugs in the management of these patients.

## Supplementary Material

Supplement

## Figures and Tables

**Table 1.** Summary of studies evaluating clinical outcomes following BTK inhibitor use in acute SARS-CoV-2 infection included in our analysis.

Study	PMCID	Type of Study	Population Size	Setting	Median age in years (range)	Malignancy	Drug (dose)	Outcome Measured
Wilkinson, T., et al.	PMC7393340	Phase I and II (randomized, unblinded, controlled)	Phase I: 60 Phase II: 126	Hospitalized, including ICU	NA	NA	Acalabrutinib (100mg twice daily for 10 days)	Resolution (Time to clinical improvement of at least 2 severity points on a 9-point category ordinal scale), discharge from hospital
Treon, S.P., et al.	PMC7243149	Case-series	6	Hospitalized and non-hospitalized, including ICU	66 (58–72)	WM	Ibrutinib (420mg daily, one patient with 140mg daily)	Hospitalization, oxygen requirements, resolution of symptoms
Scarfò, L., et al.	PMC7347048	Case-cohort	Total 190 (Ibrutinib 39; Acalabrutinib 4; Zanubrutinib 1)	Hospitalized (including ICU) and non-hospitalized	72 (48–94)	CLL, SLL	ND	Hospitalization rate, resolution of symptoms, survival
Roschewski, M., et al.	PMC7274761	Prospective Interventional	19	Hospitalized requiring oxygen support	61 (45–84)	NA	Acalabrutinib (100mg twice daily for 10–14 days)	Oxygen requirements, time to extubation
Mato, A.R., et al.	PMC7472711	Case-cohort	Total 198 (BTKi monotherapy 54; BTKinib with other agents^+^ 14)	Hospitalized (including ICU) and non-hospitalized	70.5 (38–98)	CLL	ND	Overall survival, ICU admission, oxygen Requirements
Thibaud, S., et al.	PMC7276870	Case-series	8	Hospitalized, including ICU	72 (49–88)	CLL	Ibrutinib (420 or 140 mg daily) Acalabrutinib (200 mg/daily) in one patient	Hospitalization duration, maximal oxygen requirement

**Table 2.** Risk-of-Bias Analyses of the 6 clinical studies included in our analysis.

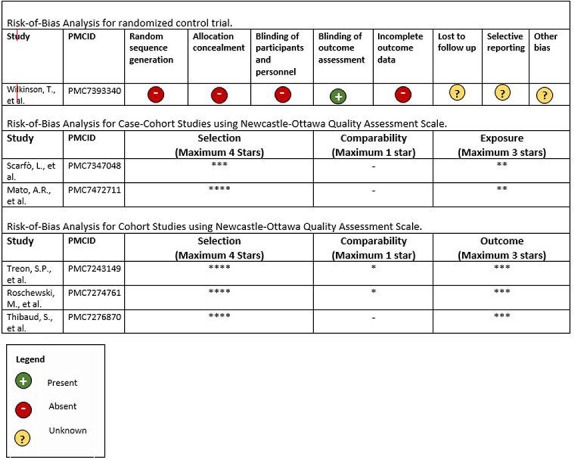

## Abbreviations

BTKinib: Bruton’s tyrosine kinase inhibitor
CLL: Chronic lymphocytic leukemia
COVID-19: Coronavirus disease 2019
JAK: Janus kinase ICU Intensive care unit
SARS-CoV-2: Severe acute respiratory syndrome coronavirus 2
WM: Waldenstrom’s macroglobulinemia
