# Supplementary material for: BTK inhibitors for Severe Acute Respiratory Syndrome Coronavirus 2 (SARS-CoV-2): A Systematic Review
Source: Res Sq. 2021 Mar 22:rs.3.rs-319342. Preprint. [Version 1] doi: 10.21203/rs.3.rs-319342/v1 (PMC8010740; doi:10.21203/rs.3.rs-319342/v1)
Supplement: Supplement [file 4198744c1b397dfa5a3af444.docx]

BTK inhibitors for Severe Acute Respiratory Syndrome Coronavirus 2 (SARS-CoV-2): A Systematic Review

# Supplemental File 1: Final search strategies used

## PubMed

**Database:** PubMed

**Vendor:** US National Library of Medicine

**Date of Search:** December 30, 2020

**Notes:** Use MeSH and keywords. Limit terms to the title and abstract fields, and the MeSH and Supplementary Concept fields.
**Limits Used:** None

**Total = 28**

(COVID[tiab] OR “SARS COV 2”[tiab] OR "COVID-19"[Mesh] OR "SARS-CoV-2"[Mesh] OR “Severe acute respiratory syndrome coronavirus 2”[tiab]) AND ("ibrutinib" [Supplementary Concept] OR "acalabrutinib" [Supplementary Concept] OR "zanubrutinib" [Supplementary Concept] OR "evobrutinib" [Supplementary Concept] OR "spebrutinib" [Supplementary Concept] OR acalabrutinib[tiab] OR branebrutinib[tiab] OR dasatinib[tiab] OR evobrutinib[tiab] OR ibrutinib[tiab] OR orelabrutinib[tiab] OR poseltinib[tiab] OR remibrutinib[tiab] OR spebrutinib[tiab] OR vecabrutinib[tiab] OR zanubrutinib[tiab] OR *“*BTK inhibitor*”[tiab] OR “Bruton s tyrosine kinase inhibitor*”[tiab] OR “Bruton Tyrosine Kinase Inhibitor*”[tiab] OR Imbruvica[tiab] OR Calquence[tiab] OR Brukinsa[tiab])

# Embase

**Database:** Embase

**Vendor:** Elsevier

**Date of Search:** December 30, 2020

**Notes:** Use EMTREE and keywords. Limit terms to the title and abstract fields, and the EMTREE field.
**Limits Used:** Source: Embase

**Total = 119**

**Total & Limited: Source = 115**

('coronavirus disease 2019'/exp OR 'Severe acute respiratory syndrome coronavirus 2'/exp OR COVID:ti,ab OR “SARS COV 2”:ti,ab OR “Severe acute respiratory syndrome coronavirus 2”:ti,ab) AND ('Bruton tyrosine kinase inhibitor'/exp OR 'acalabrutinib'/exp OR 'branebrutinib'/exp OR 'dasatinib'/exp OR 'elsubrutinib'/exp OR 'evobrutinib'/exp OR 'ibrutinib'/exp OR 'orelabrutinib'/exp OR 'poseltinib'/exp OR 'remibrutinib'/exp OR 'rilzabrutinib'/exp OR 'spebrutinib'/exp OR 'tolebrutinib'/exp OR 'vecabrutinib'/exp OR 'zanubrutinib'/exp OR acalabrutinib:ti,ab OR branebrutinib:ti,ab OR dasatinib:ti,ab OR elsubrutinib:ti,ab OR evobrutinib:ti,ab OR ibrutinib:ti,ab OR orelabrutinib:ti,ab OR poseltinib:ti,ab OR remibrutinib:ti,ab OR rilzabrutinib:ti,ab OR spebrutinib:ti,ab OR tolebrutinib:ti,ab OR vecabrutinib:ti,ab OR zanubrutinib:ti,ab OR “BTK inhibitor*”:ti,ab OR “Bruton s tyrosine kinase inhibitor*”:ti,ab OR “Bruton Tyrosine Kinase Inhibitor*”:ti,ab OR Imbruvica:ti,ab OR Calquence:ti,ab OR Brukinsa:ti,ab)

# Web of Science: Core Collection

**Database:** Web of Science: Core Collection

**Vendor:** Clarivate Analytics

**Date of Search:** December 30, 2020

**Notes:** Use keywords. Search in the Topic field (title, abstract, author keywords, Keywords Plus).
**Limits Used:** None

**Total = 24**

TS=(("coronavirus disease 2019" OR "Severe acute respiratory syndrome coronavirus 2" OR COVID OR “SARS COV 2” OR “Severe acute respiratory syndrome coronavirus 2”) AND (acalabrutinib OR branebrutinib OR dasatinib OR elsubrutinib OR evobrutinib OR ibrutinib OR orelabrutinib OR poseltinib OR remibrutinib OR rilzabrutinib OR spebrutinib OR tolebrutinib OR vecabrutinib OR zanubrutinib OR “BTK inhibitor*” OR “Bruton s tyrosine kinase inhibitor*” OR “Bruton Tyrosine Kinase Inhibitor*” OR Imbruvica OR Calquence OR Brukinsa)
